# Chemical Profile Determination and Quantitative Analysis of Components in Oryeong-san Using UHPLC-Q-Orbitrap-MS and UPLC-TQ-MS/MS

**DOI:** 10.3390/molecules28093685

**Published:** 2023-04-24

**Authors:** Seol Jang, Ami Lee, Youn-Hwan Hwang

**Affiliations:** 1 KM Convergence Research Division, Korea Institute of Oriental Medicine, Yuseong-daero 1672, Yuseong-gu, Daejeon 34054, Republic of Korea; swellseol@kiom.re.kr (S.J.); dmb01367@kiom.re.kr (A.L.); 2 Korean Convergence Medical Science Major, KIOM School, University of Science & Technology (UST), Yuseong-gu, Daejeon 34054, Republic of Korea

**Keywords:** Oryeong-san, UHPLC-Q-Orbitrap-MS, UPLC-TQ-MS/MS, quality control

## Abstract

In this study, a method to both qualitatively and quantitively analyze the components of Oryeong-san (ORS), which is composed of five herbal medicines (*Alisma orientale* Juzepzuk, *Polyporus umbellatus* Fries, *Atractylodes japonica* Koidzumi, *Poria cocos* Wolf, and *Cinnamomum cassia* Presl) and is prescribed in traditional Oriental medicine practices, was established for the first time. First, ORS components were profiled using ultra-high-performance liquid chromatography/quadrupole Orbitrap mass spectrometry, and 19 compounds were clearly identified via comparison against reference standard compounds. Subsequently, a quantitative method based on ultra-high-performance liquid chromatography coupled with triple-quadrupole tandem mass spectrometry was established to simultaneously measure the identified compounds. Nineteen compounds were accurately quantified using the multiple-reaction-monitoring mode and used to analyze the sample; we confirmed that coumarin was the most abundant compound. The method was validated, achieving good linearity (R^2^ ≤ 0.9991), recovery (RSD, 0.11–3.15%), and precision (RSD, 0.35–9.44%). The results suggest that this method offers a strategy for accurately and effectively determining the components of ORS, and it can be used for quality assessment and management.

## 1. Introduction

Oryeong-san (ORS; also known as Wulingsan in China and Gorei-san in Japan) is a traditional Korean prescription manufactured in Sanghanron; it is composed of five herbal medicines (*Alisma orientale* Juzepzuk, *Polyporus umbellatus* Fries, *Atractylodes japonica* Koidzumi, *Poria cocos* Wolf, and *Cinnamomum cassia* Presl at the ratio 5:3:3:3:2) [1,2]. ORS is prescribed to promote diuresis, reduce edema, and improve water metabolism in the body [3,4]. In addition, it is widely used in the treatment of renal diseases, and has been reported to improve renal functioning through antihypertensive and antidiabetic effects [5,6,7]. Clinical studies have also reported that ORS is effective in preventing calcium oxalate nephrolithiasis [8]. In addition, ORS was found to reduce gastrointestinal adverse reactions in patients taking selective serotonin reuptake inhibitors (SSRIs), and it was proven to reduce hematoma in patients with chronic subdural hematoma (CSDH) [9,10]. Traditional Oriental medicines (TOMs), which include prescriptions such as ORS, are widely used to prevent diseases, owing to their efficacy and low toxicity [11]. When evaluating the quality of TOMs, the components of various herbal medicines are selected as evaluation indicators; however, these approaches primarily focus on individual herbal medicines. If prescriptions are composed of two or more herbal medicines, their chemical properties may differ from those of a single herbal medicine; this makes it difficult to accurately reflect their characteristics in prescription quality evaluations [12,13,14].

According to Korean Pharmacopoeia [15], the quality control of each herbal medicine and ORS except *C. cassia* is mainly managed by thin-layer chromatographic analysis. The five individual herbal medicine components in ORS have been reported as follows: triterpenoids (e.g., alisol A) from *A. orientale* [16], steroids (e.g., polyporusterone A) from *P. umbellatus* [17], sesquiterpenoids (e.g., atractyloside A) from *A. japonica* [18], triterpenoids (e.g., 16α-hydroxytrametenolic acid) from *P. cocos* [19], coumarins (e.g., coumarin), and flavonoids (e.g., procyanidin B1) from *C. cassia* [20]. In addition, studies have simultaneously determined the components of ORS using high-performance liquid chromatography and liquid chromatography–mass spectrometry (LC-MS) for ORS quality control [1,21,22]. However, these studies are limited to the quantitative analysis of several major components or the screening-based qualitative analysis of all components. Therefore, it is necessary to establish an appropriate analytical method that can simultaneously identify components and determine their contents to facilitate accurate quality control of the ORS.

LC-MS methods are widely used to identify and characterize the chemical compositions of various TOMs, as well as their related preparations [23,24,25]. These methods can facilitate comprehensive chemical profiling; in particular, mass spectrometry using an Orbitrap analyzer offers ion information with low mass errors, thereby facilitating rapid component identification [14]. In addition, triple-quadrupole mass spectrometry (TQ-MS/MS) using multiple-reaction monitoring (MRM) is a highly sensitive and powerful quantitative method offering high throughput [26,27].

Therefore, in this study, an analysis method based upon ultra-high-performance liquid chromatography/quadrupole Orbitrap mass spectrometry (UHPLC-Q-Orbitrap-MS) was established to identify the components of ORS, and 19 compounds were identified. For the simultaneous quantitative analysis of the identified compounds, an ultra-performance liquid chromatography coupled with a triple-quadrupole tandem mass spectrometry (UPLC-TQ-MS/MS) analysis method using the MRM mode was established and applied for content analysis.

## 2. Results and Discussion

### 2.1. Identification of Compounds in ORS via UHPLC-Q-Orbitrap-MS

**Figure 1:**
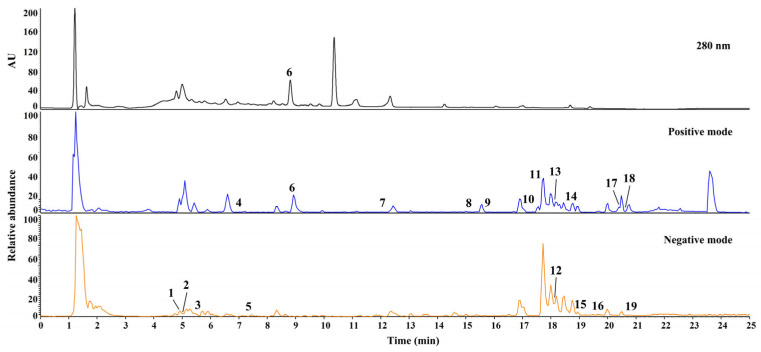
UHPLC-Q-Orbitrap-MS base peak ion chromatograms of ORS extract. The ID numbers of the various types of phytochemicals are listed in Table 1.

Qualitative analysis was performed to identify the chemical compounds in ORS; to this end, UHPLC-Q-Orbitrap-MS was used. The chemical compounds in ORS were identified by comparing the retention times and mass spectra of the reference standard compounds, and 19 compounds were identified. The base peak chromatograms of the ORS in both positive and negative ion modes are shown in Figure 1, and detailed information regarding the identified compounds is listed in Table 1. Injection peak was found around 1.5 min, and this peak may be composed of the various eluents of sugars, amino acids, and so on (Figure 1). The apparent peak at 10.4 min, which were only found in UV chromatogram, was not sufficient to identify a certain phytochemical with low intensity and less MS_2_ fragmentation pattern (Figure 1, upper panel). In this regard, the quantitative analysis of ORS was performed except for the above-mentioned peaks.

Among the chemical compounds identified in ORS, 16 compounds were identical to those reported in previous studies; these are as follows: alisol A, alisol A 24-acetate, alisol B, alisol B 23-acetate, alisol C, and alisol C 23-acetate from *A. orientale*; polyporusterone A from *P. umbellatus*; atractyloside A, atractylenolide Ⅰ, atractylenolide Ⅱ, and atractylenolide Ⅲ from *A. japonica*; 16α-hydroxytrametenolic acid, 3-*O*-acetyl-16α- hydroxytrametenolic acid, and pachymic acid from *P. cocos*; and procyanidin B2 and coumarin from *C. cassia* [5,22,28]. As such, some studies on the pharmacological activities of the compounds identified in ORS have been reported. It has been reported that procyanidin B1, procyanidin B2, and rosavin have antioxidant activities, and coumarin and polyporusterone A have various activities, such as anti-inflammatory, antioxidant, and anticancer activities [17,29,30,31]. Atractyloside A, a compound of *A. japonica*, and other compounds were also found to have anti-inflammatory activity [18]. In addition, studies have reported that pachymic acid and other compounds that are components of *P. cocos*, and alisol A and other compounds of *A. orientale*, have anti-inflammatory and anticancer effects [32,33].

### 2.2. Quantitative Analysis of Compounds in ORS Using UPLC-TQ-MS/MS

**Figure 2:**
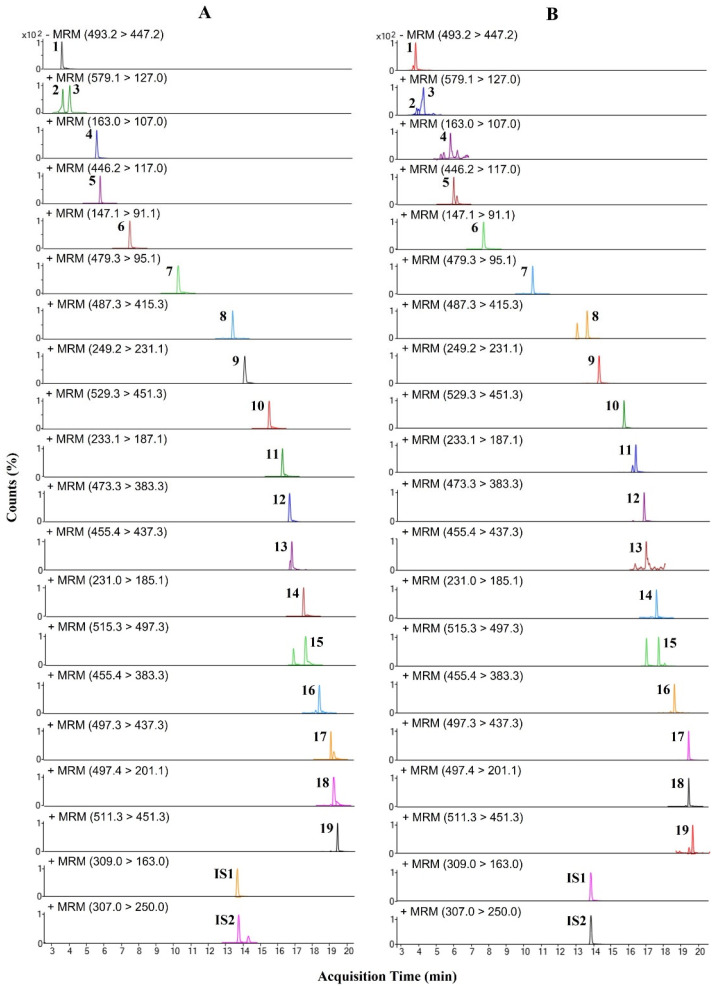
UPLC-TQ-MS/MS chromatograms in MRM mode for (**A**) mixed-reference solution and (**B**) ORS extract.

The UPLC-TQ-MS/MS method (in the MRM mode) was used to quantify the compounds in ORS, and 19 compounds were simultaneously detected within 20 min. The MRM mode allows for highly specific and sensitive analyses [34]. A single standard solution of each analyte was injected to investigate the ion pairs (consisting of precursor and product ions in both positive and negative ion modes). Atractyloside A was determined in the negative ion mode, and all remaining compounds were determined in the positive ion mode. For all analytes (including the internal standard, IS), the MRM parameters (including the selected MRM pairs and collision energies) were optimized (the details are listed in Table 2). The chromatograms of the 19 compounds and IS, as obtained in the MRM mode, are shown in Figure 2.

The same precursor ion (*m*/*z* 579.1) was selected for procyanidins B1 and B2, and a characteristic fragment ion was produced at *m*/*z* 127.0; this was selected as the product ion for each compound [35]. Umbelliferone and coumarin generated precursor ions in the form of [M + H]^+^ at *m*/*z* 163.0 and *m*/*z* 147.1, respectively; both compounds formed product ions in the form of [M + H − 2CO]^+^. Umbelliferone formed a product ion at *m*/*z* 107.0, and coumarin formed a product ion at *m*/*z* 91.1 as a result of the loss of both CO_2_ (*m*/*z* 44) and C (*m*/*z* 12) [36,37,38]. The precursor ions of alisol A and alisol A 24-acetate were formed at *m*/*z* 473.3 and *m*/*z* 515.3, respectively, in the form of [M + H − H_2_O]^+^; however, in the case of the product ion, alisol A was formed at *m*/*z* 383.3 in the form of [M + H − H_2_O − C_4_H_10_O_2_]^+^, and alisol A 24-acetate was formed at *m*/*z* 497.3 in the form of [M + H − 2H_2_O]^+^. Similarly, alisol B produced a precursor ion at *m*/*z* 455.4 in the form of [M + H − H_2_O]^+^ and a product ion at *m*/*z* 383.3 in the form of [M + H − H_2_O − C_4_H_8_O]^+^. In addition, the precursor ions of alisol C and alisol C 23-acetate were produced in the form of [M + H]^+^ at *m*/*z* 487.3 and *m*/*z* 529.3, respectively, and alisol C formed a product ion in the form of [M + H − C_4_H_8_O]^+^ at *m*/*z* 415.3. For alisol C 23-acetate, the loss of HAc at C-23 and the loss of H_2_O (18 Da) occurred simultaneously to generate a product ion in the form of [M + H − Hac − H_2_O]^+^ at *m*/*z* 451.3 [39,40]. The lactone components, atractylenolide I, II, and III, produced precursor ions in the form of [M + H]^+^ at *m*/*z* 231.0, *m*/*z* 233.1, and *m*/*z* 249.2, respectively. Atractylenolide I and atractylenolide II produced product ions in the form of [M + H − H_2_O − CO]^+^ at *m*/*z* 185.1 and *m*/*z* 187.1, respectively, via the simultaneous loss of H_2_O and CO groups. Unlike the other two compounds, atractylenolide III formed a product ion in the form of [M + H − H_2_O]^+^ at *m*/*z* 231.1, owing to the loss of H_2_O molecules [41,42]. 16α-hydroxytrametenolic acid and 3-*O*-acetyl-16α-hydroxytrametenolic acid generated precursor ions at *m*/*z* 455.4 and *m*/*z* 497.3, respectively, in the form of [M + H − H_2_O]^+^; both compounds formed product ions at *m*/*z* 437.3: 16α-hydroxytrametenolic acid in the form of [M + H − 2H_2_O]^+^ and 3-*O*-acetyl-16α-hydroxytrametenolic acid in the form of [M + H − H_2_O − CH_3_COOH]^+^, respectively [43]. Similarly, pachymic acid generated a precursor ion in the form of [M + H − 2H_2_O]^+^ at *m*/*z* 511.3 and then formed a product ion in the form of [M + H − H_2_O − CH_3_COOH]^+^ at *m*/*z* 451.3, owing to the loss of the AcOH moiety [44].

### 2.3. Method Validation

Calibration curves for all analytes were plotted against the concentrations of the standard solutions, and the linearity of the analytical method was evaluated using the correlation coefficient of each calibration curve. The correlation coefficient of the calibration curve exhibited good linearity (>0.9991) within the test range. The lower limit of quantitation (LLOQ) was within the range of 0.01–0.20 ng/mL for all compounds. Therefore, it was confirmed that the established method provides a sensitive quantitative analysis of ORS compounds. Detailed information for the regression equation, correlation coefficient (R^2^), linear range, and LLOQ is listed in Table 3.

Recovery tests were performed by spiking mixed standard solutions of three different concentrations with a known quantity of the sample. As shown in Table 4, the recovery values of the 19 compounds were 89.32–110.32%, and the relative standard deviation (RSD) was less than 3.15, indicating that the accuracy of the quantification method was good.

The precision and accuracy were validated by mixing standard solutions at three concentrations, as shown in Table 5. Intra- and inter-day tests were performed by analyzing a sample prepared six times within the same day and over three consecutive days, respectively. The precision of the method was expressed in terms of the RSD; the intra-day value was less than 7.32%, the inter-day value was less than 9.44%, and the accuracy of the intra- and inter-day values varies as 89.17–112.34% and 88.86–110.20%, respectively.

These results indicate that the established analytical method based on UPLC-TQ-MS/MS can accurately and efficiently quantify the 19 constituent compounds of ORS.

### 2.4. Sample Analysis

The established UPLC-TQ-MS/MS method was used to simultaneously determine 19 compounds in 3 batches of ORS. The contents of all compounds, as obtained from the calibration curves calculated via the IS method, are shown in Table 6.

Among the 19 compounds measured, the coumarin content was highest (at 7.391–7.683 ng/g) in the 3 batches of ORS; these results resemble those of previously reported studies [2]. In addition to coumarin, the contents of alisol B 23-acetate, atractyloside A, and atractylenolide III were higher in ORS than in the other compounds. These four compounds are derived from *C. cassia*, *A. orientale*, and *A. japonica* (three of the single-herb medicines constituting ORS) and they have been reported as major compounds in previous studies into these individual herbal medicines [18,20,45]. The UPLC-TQ-MS/MS method established in our study was successfully applied to simultaneously quantify the 19 compounds, demonstrating the method’s suitability for component analysis of ORS.

## 3. Materials and Methods

### 3.1. Materials and Reagents

The reference standards used in this study were atractyloside A, procyanidin_B1, procyanidin B2, umbelliferon, rosavin, coumarin, alisol C, atractylenolide III, alisol C 23-acetate, atractylenolide II, alisol A, 16α-hydroxytrametenolic acid, atractylenolide I, alisol A 24-acetate, alisol B, 3-*O*-acetyl-16α-hydroxytrametenolic acid, alisol B 23-acetate, and pachymic acid; these were purchased from ChemFaces (Wuhan, China). Polyporusterone A was purchased from Chem-Norm Biotech (Wuhan, China), and warfarin, an IS, was purchased from Sigma-Aldrich (St. Louis, MO, USA). All reference standards were used with a purity of 98% or higher. MS-grade methanol, acetonitrile, water, and formic acid were purchased from Thermo Fisher Scientific (Waltham, MA, USA). The five herbal medicines (*A. orientale*, *P. umbellatus*, *A. japonica*, *P. cocos*, and *P. cocos*) were purchased from Kwangmyungdang Pharmaceutical (Ulsan, Republic of Korea). Each raw herbal medicine was deposited at the KM Convergence Research Division of the Korea Institute of Oriental Medicine (specimen No. TDC-01, TDC-04, and TDC-06–08).

### 3.2. Preparation of ORS

ORS extracts were prepared using the method described in a previous study [46]. In total, 5 herbal medicines were combined in the ratios shown in Table 7, and 10 times their total weight of water was added, followed by reflux extraction at 100 °C for 3 h. The extracted water was then filtered and concentrated under reduced pressure using a rotary evaporator. The concentrated aqueous extract was freeze-dried, and the obtained powder sample (yield: 20.3%) was used for analyses.

### 3.3. Preparation of Standard and Sample Solutions

Nineteen standard compounds and one IS (warfarin) were individually dissolved in methanol to prepare stock solutions. The stock solutions were prepared by mixing aliquots for each solution. Working solutions containing 19 compounds were diluted in methanol to prepare a set of appropriate concentrations. The IS concentration was kept consistent in each sample at 5 ng/mL. Quality control (QC) samples were used for method validation and prepared at high, medium, and low concentrations using the method described above. ORS powder (20 mg) was extracted using methanol in an ultrasonic bath for 30 min. The extract was centrifuged at 12,500 rpm for 15 min and analyzed.

### 3.4. UHPLC-Q-Orbitrap-MS Conditions for Qualitative Analysis

The UHPLC-Q-Orbitrap-MS method was performed on a Dionex UltiMate 3000 system equipped with a Thermo Q-Exactive mass spectrometer (Thermo Fisher Scientific, Waltham, MA, USA). The analytical conditions for determining the chemical compounds in the ORS matched those of previously reported analytical methods [46]. In brief, the separation was performed on an C18 column (100 × 2.1 mm, 1.7 µm, Acquity BEH C18, Waters, Milford, MA, USA), and a mobile phase (a gradient mixture of 0.1% formic acid in water (A) and acetonitrile (B)) was used. All samples were analyzed in the positive and negative ion conversion modes, and the mass scan was performed in the range 100–1500 *m*/*z*. Full scan and MS/MS scan data were acquired at resolutions of 70,000 full width at half maximum and 17,500 in both positive and negative modes, respectively. Xcalibur v.3.0 (Thermo Fisher Scientific, Waltham, MA, USA) was used to acquire and analyze the data.

### 3.5. UPLC-TQ-MS/MS Conditions for Quantitative Analysis

Quantitative analyses were conducted using an Agilent 1290 Infinity II system interfaced with an Agilent 6495C triple-quadrupole mass spectrometer (Agilent Technologies, Santa Clara, CA, USA). Ionization was performed using a jet-stream electrospray ionization source. The operating conditions for chromatographic separation and mass spectrometric detection were determined using previously reported methods [46]. MRM was performed to quantify the 19 ORS compounds, and the MRM transitions and collision energy values were optimized for each compound (Table 2). All MRM data were acquired and processed using the Agilent MassHunter workstation quantitative analysis software (version 10.1).

### 3.6. Method Validation of Quantitative Analysis

The UPLC-TQ-MS/MS method used to quantitatively analyze the 19 ORS compounds was validated using linearity, recovery, precision, and accuracy parameters [47]. Linearity was evaluated by constructing a calibration curve for each compound, using the peak area ratio between the analyte concentration and IS. The LLOQ was defined as the lowest concentration in the standard curve that could be measured with acceptable accuracy and precision, and the limit of quantification (LOQ) was determined at a signal to noise (S/N) ratio of 10. To investigate the recovery, different concentrations (high, medium, and low) for each compound were added to the ORS samples. Recovery was evaluated by comparing the spiked and detected quantities of each analyte. Intra- and inter-day variations were performed to evaluate the method precision. QC samples were prepared at three concentration levels, and precision was determined using the relative standard deviation calculated from the measured concentrations. To evaluate intra-day precision, QC samples were measured six times within one day; to evaluate inter-day precision, the same samples were measured on three consecutive days.

## 4. Conclusions

In this study, the qualitative and quantitative analysis methods UHPLC-Q-Orbitrap-MS and UPLC-TQ-MS/MS, respectively, were applied to comprehensively analyze ORS components. Chemical profiling of ORS was performed using the developed UHPLC-Q-Orbitrap-MS analysis method, and 19 compounds were identified via comparison with reference standard compounds. The 19 identified compounds were simultaneously quantified within 20 min using an established MRM-mode quantitative analysis method; this was successfully applied for practical sample analysis. These results facilitate the qualitative and quantitative analysis of ORS constituents, in turn facilitating the accurate chemical identification and simultaneous determination of compounds. It also aids in the routine analysis of ORS and the identification of biologically active substances, suggesting that it can be effectively applied for overall quality control.

## Figures and Tables

**Table 1:** Characterization of chemical constituents of ORS using UHPLC-Q-Orbitrap-MS.

No.	Identification	Rt (min)	Formula	Adduct	Predicted (*m*/*z*)	Measured (*m*/*z*)	Error (ppm)	MS/MS (*m*/*z*)
1	Atractyloside A	4.95	C_21_H_36_O_10_	[M + HCO_2_]^−^	493.2290	493.2293	−0.02	447.2245, 285.1714, 89.0229
2	Procyanidin B1	5.05	C_30_H_26_O_12_	[M − H]^−^	577.1351	577.1352	−0.34	407.0771, 289.0720 125.0230
3	Procyanidin B2	5.62	C_30_H_26_O_12_	[M − H]^−^	577.1351	577.1351	−0.44	407.0774, 289.0721 125.0231
4	Umbelliferone	7.12	C_9_H_6_O_3_	[M + H]^+^	163.0390	163.0388	−0.84	163.0388
5	Rosavin	7.40	C_20_H_28_O_10_	[M + HCO_2_]^−^	473.1664	473.1661	−1.23	293.0878, 89.0228
6	Coumarin	9.09	C_9_H_6_O_2_	[M + H]^+^	147.0441	147.0440	−0.44	147.0439, 103.0546
7	Polyporusterone A	12.02	C_28_H_46_O_6_	[M + H]^+^	479.3365	479.3365	−0.40	95.0861
8	Alisol C	15.16	C_30_H_46_O_5_	[M + H]^+^	487.3418	487.3417	−0.18	415.2840
9	Atractylenolide III	15.79	C_15_H_2_0O_3_	[M + H]^+^	249.1485	249.1483	−0.88	231.1379, 163.0753
10	Alisol C 23-acetate	17.07	C_32_H_48_O_6_	[M + H]^+^	529.3524	529.3526	0.44	451.3205
11	Atractylenolide II	17.69	C_15_H_2_0O_2_	[M + H]^+^	233.1536	233.1535	−0.57	233.1535, 215.1431 187.1481, 151.0754
12	Alisol A	18.12	C_30_H_50_O_5_	[M + HCO_2_]^−^	535.3640	535.3643	−0.01	471.3499
13	16α-Hydroxytrametenolic acid	18.13	C_30_H_48_O_4_	[M + H]^+^	473.3625	473.3625	−0.03	437.3433, 295.2415
14	Atractylenolide I	18.79	C_15_H_18_O_2_	[M + H]^+^	231.1380	231.1379	−0.27	231.1379
15	Alisol A 24-acetate	18.93	C_32_H_52_O_6_	[M + HCO_2_]^−^	577.3746	577.3748	−0.02	169.0408, 59.0122
16	Alisol B	19.82	C_30_H_48_O_4_	[M + HCO_2_]^−^	517.3535	517.3535	−0.48	241.4872, 100.0714
17	3-*O*-Acetyl-16α-hydroxytrametenolic acid	20.42	C_32_H_50_O_5_	[M + H]^+^	515.3731	515.3730	−0.09	437.3416, 295.2421 133.0860, 89.0603
18	Alisol B 23-acetate	20.62	C_32_H_50_O_5_	[M + H]^+^	515.3731	515.3729	−0.33	339.2672, 151.1116 97.0653
19	Pachymic acid	20.82	C_33_H_52_O_5_	[M − H]^−^	527.3742	527.3740	−0.90	527.3741

**Table 2:** Optimized MRM parameters of the 19 compounds in the UPLC-TQ-MS/MS.

No.	Compound	Rt (min)	MW	MRM Transition (*m*/*z*)	Collision Energy (V)
1	Atractyloside A	3.63	448.5	493.2 → 447.2	14
2	Procyanidin B1	3.68	578.5	579.1 → 127.0	30
3	Procyanidin B2	4.09	578.5	579.1 → 127.0	30
4	Umbelliferone	5.67	162.1	163.0 → 107.0	22
5	Rosavin	5.84	428.4	446.2 → 117.0	14
6	Coumarin	7.55	146.1	147.1 → 91.1	26
7	Polyporusterone A	10.34	478.7	479.3 → 95.1	30
8	Alisol C	13.48	486.7	487.3 → 415.3	18
9	Atractylenolide III	14.18	248.3	249.2 → 231.1	10
10	Alisol C 23-acetate	15.59	528.7	529.3 → 451.3	18
11	Atractylenolide II	16.28	232.3	233.1 → 187.1	14
12	Alisol A	16.62	490.7	473.3 → 383.3	10
13	16α-Hydroxytrametenolic acid	16.71	472.7	455.4 → 437.3	18
14	Atractylenolide I	17.40	230.3	231.0 → 185.1	18
15	Alisol A 24-acetate	17.50	532.8	515.3 → 497.3	10
16	Alisol B	18.41	472.7	455.4 → 383.3	10
17	3-*O*-Acetyl-16α-hydroxytrametenolic acid	19.06	514.7	497.3 → 437.3	18
18	Alisol B 23-acetate	19.24	514.7	497.4 → 201.1	22
19	Pachymic acid	19.45	528.8	511.3 → 451.3	18
IS1	Warfarin	13.64	307.1	309.0 → 163.0	14
IS2	Warfarin	13.64	307.1	307.0 → 250.0	22

**Table 3:** Calibration curves, linear range, and lower limit of quantification (LLOQ) for the 19 compounds.

No.	Compound	Calibration Curves	R^2^	Linear Range (ng/mL)	LLOQ (ng/mL)
1	Atractyloside A	y = 1.5739x + 0.0418	0.9995	0.10–25.00	0.10
2	Procyanidin B1	y = 0.2186x − 0.0094	0.9991	0.10–12.50	0.10
3	Procyanidin B2	y = 0.2277x − 0.0083	0.9994	0.10–12.50	0.10
4	Umbelliferone	y = 3.7074x − 0.0012	0.9996	0.01–3.13	0.01
5	Rosavin	y = 2.4065x − 0.0108	0.9993	0.02–3.13	0.02
6	Coumarin	y = 3.9901x + 0.1995	0.9994	0.10–50.00	0.10
7	Polyporusterone A	y = 0.9290x − 0.0015	0.9998	0.10–25.00	0.10
8	Alisol C	y = 1.2274x − 0.0099	0.9993	0.05–6.25	0.05
9	Atractylenolide III	y = 2.9593x − 0.0390	0.9996	0.10–25.00	0.10
10	Alisol C 23-acetate	y = 5.9883x + 0.0233	0.9992	0.01–3.13	0.01
11	Atractylenolide II	y = 7.2963x + 0.0235	0.9995	0.01–3.13	0.01
12	Alisol A	y = 1.4905x − 0.0296	0.9995	0.10–25.00	0.10
13	16α-Hydroxytrametenolic acid	y = 0.6745x − 0.0043	0.9991	0.02–3.13	0.02
14	Atractylenolide I	y = 3.8661x + 0.0091	0.9993	0.01–3.13	0.01
15	Alisol A 24-acetate	y = 1.2214x − 0.0693	0.9993	0.20–25.00	0.20
16	Alisol B	y = 0.2495x − 0.0075	0.9992	0.20–25.00	0.20
17	3-*O*-Acetyl-16α-hydroxytrametenolic acid	y = 0.5743x − 0.0036	0.9994	0.02–3.13	0.02
18	Alisol B 23-acetate	y = 0.5967x + 0.0013	0.9992	0.05–12.50	0.05
19	Pachymic acid	y = 1.1861x − 0.0156	0.9991	0.05–6.25	0.05

**Table 4:** Recovery of the 19 compounds in ORS.

No.	Compound	Spiked Concentration (ng/mL)	Measured Concentration (ng/mL)	Recovery (%)	RSD (%)
1	Atractyloside A	10.671	10.160	95.21	1.09
		4.421	4.349	98.39	0.90
		2.858	2.891	101.17	1.67
2	Procyanidin B1	4.351	3.998	91.89	1.18
		1.226	1.245	101.60	1.29
		0.445	0.468	105.29	1.13
3	Procyanidin B2	4.458	3.982	89.32	0.91
		1.333	1.255	94.11	2.38
		0.552	0.550	99.60	1.33
4	Umbelliferone	1.051	1.011	96.20	0.81
		0.270	0.272	100.93	1.30
		0.075	0.069	92.96	1.73
5	Rosavin	1.223	1.215	99.39	1.09
		0.442	0.438	99.09	0.76
		0.246	0.238	96.82	1.64
6	Coumarin	11.404	10.354	90.79	0.97
		5.154	5.686	110.32	0.47
		3.592	3.713	103.37	0.91
7	Polyporusterone A	8.429	7.874	93.41	1.02
		2.179	2.154	98.86	0.66
		0.617	0.568	92.02	0.61
8	Alisol C	2.322	2.281	98.22	1.20
		0.759	0.756	99.55	1.25
		0.369	0.363	98.44	0.63
9	Atractylenolide III	10.491	10.006	95.37	0.83
		4.241	4.164	98.19	1.08
		2.678	2.629	98.14	0.70
10	Alisol C 23-acetate	2.312	2.359	102.03	0.56
		1.531	1.590	103.90	0.73
		1.335	1.456	109.02	0.99
11	Atractylenolide II	1.482	1.463	98.71	2.57
		0.700	0.716	102.23	1.98
		0.505	0.537	106.31	3.15
12	Alisol A	8.927	8.475	94.94	0.44
		2.677	2.521	94.21	0.51
		1.114	1.050	94.28	0.87
13	16α-Hydroxytrametenolic acid	1.067	1.058	99.18	1.32
		0.286	0.280	97.95	1.53
		0.091	0.084	92.76	3.07
14	Atractylenolide I	1.115	1.036	92.90	1.81
		0.334	0.334	99.95	1.47
		0.139	0.146	105.17	0.48
15	Alisol A 24-acetate	8.635	7.766	89.94	0.11
		2.385	2.214	92.84	0.65
		0.822	0.776	94.43	0.43
16	Alisol B	8.966	8.620	96.14	1.17
		2.716	2.517	92.67	1.71
		1.153	1.055	91.51	2.49
17	3-*O*-Acetyl-16α-hydroxytrametenolic acid	1.094	1.069	97.66	1.06
		0.313	0.308	98.29	0.65
		0.118	0.107	90.68	2.20
18	Alisol B 23-acetate	6.990	6.709	95.98	1.05
		3.865	3.890	100.65	1.56
		3.084	3.171	102.83	1.30
19	Pachymic acid	2.186	2.092	95.68	1.36
		0.624	0.603	96.66	1.11
		0.233	0.227	97.36	2.88

**Table 5:** Precision and accuracy of the 19 compounds in ORS.

No.	Compound	Concentration (ng/mL)	Intra-Day		Inter-Day	
			Precision (%)	Accuracy (%)	Precision (%)	Accuracy (%)
1	Atractyloside A	16.67	1.39	105.13	3.01	102.02
		4.17	2.46	105.61	3.97	101.06
		1.04	2.42	108.63	7.03	101.57
2	Procyanidin B1	8.33	1.73	96.24	2.53	96.19
		2.08	7.32	91.29	0.40	90.99
		0.52	1.72	94.25	9.44	97.56
3	Procyanidin B2	8.33	1.43	96.55	1.99	98.25
		2.08	1.52	89.43	1.82	88.86
		0.52	4.31	92.04	0.97	91.15
4	Umbelliferone	2.08	1.14	104.94	1.01	106.14
		0.52	0.77	102.78	2.25	105.50
		0.13	0.87	95.94	4.41	95.53
5	Rosavin	2.08	0.72	96.17	4.09	98.06
		0.52	1.35	95.38	2.95	96.38
		0.13	0.98	99.75	3.51	98.32
6	Coumarin	16.67	0.44	103.09	3.35	107.23
		4.17	1.52	108.42	1.66	107.39
		1.04	0.95	108.28	2.40	106.47
7	Polyporusterone A	16.67	0.65	107.98	6.53	105.20
		4.17	0.59	104.15	5.62	102.74
		1.04	0.97	93.65	3.49	97.52
8	Alisol C	4.17	1.23	101.40	0.35	101.56
		1.04	1.36	104.58	1.65	103.01
		0.26	1.28	104.09	4.13	103.30
9	Atractylenolide III	16.67	1.36	109.94	2.78	106.52
		4.17	0.77	106.08	1.32	104.49
		1.04	0.64	90.99	1.70	92.68
10	Alisol C 23-acetate	2.08	0.93	108.62	6.55	104.88
		0.52	0.55	108.14	3.27	106.80
		0.13	1.14	98.57	1.79	99.63
11	Atractylenolide II	2.08	1.33	109.62	3.45	106.47
		0.52	3.25	112.34	2.87	110.20
		0.13	2.62	99.07	2.11	98.06
12	Alisol A	16.67	0.85	106.82	3.93	103.79
		4.17	0.96	102.06	4.29	99.09
		1.04	0.94	92.37	2.65	94.02
13	16α-Hydroxytrametenolic acid	2.08	2.29	100.72	9.09	101.95
		0.52	2.67	89.17	6.92	93.24
		0.13	3.91	98.91	3.27	97.03
14	Atractylenolide I	2.08	0.62	107.66	1.80	105.46
		0.52	1.14	109.94	4.81	107.22
		0.13	2.57	93.70	1.52	95.15
15	Alisol A 24-acetate	16.67	0.76	111.72	6.84	108.29
		4.17	0.83	99.21	5.50	97.96
		1.04	1.52	93.60	3.05	94.06
16	Alisol B	16.67	1.48	109.18	5.11	106.96
		4.17	1.33	101.20	4.18	99.03
		1.04	1.23	94.37	1.12	93.73
17	3-*O*-Acetyl-16α-hydroxytrametenolic acid	2.08	1.16	93.66	3.90	96.65
		0.52	1.75	101.84	5.62	95.74
		0.13	1.09	100.28	2.29	98.15
18	Alisol B 23-acetate	8.33	1.06	110.55	7.93	106.71
		2.08	3.74	111.31	8.95	106.38
		0.52	2.38	92.11	1.83	93.99
19	Pachymic acid	4.17	1.56	95.04	7.22	98.00
		1.04	0.98	94.44	3.02	92.49
		0.26	1.04	95.46	2.37	98.11

**Table 6:** Contents of the 19 compounds in 3 batches of ORS.

No.	Compound	ORS-1			ORS-2			ORS-3		
		Mean (ng/g)	SD	CV (%)	Mean (ng/g)	SD	CV (%)	Mean (ng/g)	SD	CV (%)
1	Atractyloside A	3.697	0.057	1.549	3.511	0.060	1.722	3.699	0.043	1.171
2	Procyanidin B1	0.265	0.004	1.320	0.263	0.001	0.349	0.264	0.004	1.514
3	Procyanidin B2	0.550	0.013	2.396	0.525	0.010	1.933	0.554	0.020	3.542
4	Umbelliferone	0.022	0.000	2.100	0.023	0.001	2.503	0.023	0.001	2.341
5	Rosavin	0.276	0.004	1.530	0.264	0.006	2.403	0.280	0.001	0.226
6	Coumarin	7.611	0.066	0.873	7.391	0.100	1.348	7.683	0.082	1.070
7	Polyporusterone A	0.239	0.004	1.528	0.231	0.003	1.222	0.242	0.004	1.574
8	Alisol C	0.395	0.004	1.112	0.382	0.007	1.843	0.400	0.006	1.390
9	Atractylenolide III	3.208	0.090	2.811	3.117	0.050	1.601	3.277	0.056	1.701
10	Alisol C 23-acetate	2.540	0.016	0.645	2.437	0.015	0.606	2.573	0.028	1.079
11	Atractylenolide II	0.749	0.009	1.256	0.726	0.015	2.056	0.770	0.014	1.857
12	Alisol A	1.027	0.013	1.226	0.994	0.009	0.871	1.045	0.014	1.353
13	16α-Hydroxytrametenolic acid	0.070	0.003	3.847	0.068	0.003	4.264	0.070	0.003	4.123
14	Atractylenolide I	0.108	0.003	2.979	0.104	0.002	1.737	0.108	0.004	3.866
15	Alisol A 24-acetate	0.689	0.004	0.539	0.680	0.008	1.150	0.694	0.005	0.690
16	Alisol B	1.120	0.036	3.225	1.078	0.018	1.647	1.134	0.024	2.095
17	3-*O*-Acetyl-16α-hydroxytrametenolic acid	0.102	0.001	1.420	0.099	0.002	1.923	0.101	0.005	4.674
18	Alisol B 23-acetate	4.406	0.092	2.084	4.181	0.226	5.403	4.433	0.071	1.602
19	Pachymic acid	0.212	0.003	1.316	0.206	0.005	2.328	0.215	0.004	2.037

**Table 7:** Composition of ORS.

Scientific Name	Scientific Name	Weight Ratio
Alismatis Rhizoma	*Alisma orientale* Juzepzuk	5.0
Polyporus	*Polyporus umbellatus* Fries	3.0
Atractylodis Rhizoma Alba	*Atractylodes japonica* Koidzumi	3.0
Poria Sclerotium	*Poria cocos* Wolf	3.0
Cinnamomi Cortex	*Cinnamomum cassia* Presl	2.0

## Data Availability

The data presented in this study are available upon request from the corresponding author.

## References

[1] Lee J., Weon J.B., Lee B., Yun B.-R., Eom M.R., Ma C.J. (2013). Simultaneous determination of six components in the traditional herbal medicine ‘Oryeongsan’ by HPLC-DAD and LC-MS/MS. Nat. Prod. Sci..

[2] Lee M.Y., Seo C.S., Kim J.Y., Shin H.K. (2015). Genotoxicity evaluation of Oryeong-san water extract using in vitro and in vivo tests. BMC Complement. Altern. Med..

[3] He L., Rong X., Jiang J.M., Liu P.Q., Li Y. (2008). Amelioration of anti-cancer agent adriamycin-induced nephrotic syndrome in rats by Wulingsan (Gorei-San), a blended traditional Chinese herbal medicine. Food Chem. Toxicol..

[4] Kim J.-H., Shin H.-K. (2012). Analysis of biological experiment on Oryeong-san (Wuling-san). J. Intern. Korean Med..

[5] Kiga C., Goto H., Sakurai H., Hayashi K., Hikiami H., Shimada Y., Saiki I. (2008). Effects of traditional Japanese (Kampo) medicines (orengedokuto, goreisan and shichimotsukokato) on the onset of stroke and expression patterns of plasma proteins in spontaneously hypertensive stroke-prone rats. J. Trad. Med..

[6] Kim S.J., Leem H.H., Nam W.H., Son S.M., Choi H.M., Kim M.J., Kim J.O., Lee H.D. (2020). Effect of anti-inflammation on Oryeong-san formulation for Mix extract tablet. J. Physiol. Pathol. Korean Med..

[7] Liu I.M., Tzeng T.F., Liou S.S., Chang C.J. (2009). The amelioration of streptozotocin diabetes-induced renal damage by Wu-Ling-San (Hoelen Five Herb Formula), a traditional Chinese prescription. J. Ethnopharmacol..

[8] Lin E., Ho L., Lin M.S., Huang M.H., Chen W.C. (2013). Wu-Ling-San formula prophylaxis against recurrent calcium oxalate nephrolithiasis—A prospective randomized controlled trial. Afr. J. Tradit. Complement. Altern. Med..

[9] Yamada K., Yagi G., Kanba S. (2003). Effectiveness of Gorei-san (TJ-17) for treatment of SSRI-induced nausea and dyspepsia: Preliminary observations. Clin. Neuropharmacol..

[10] Nakao J., Marushima A., Fujita K., Fujimori H., Mashiko R., Kamezaki T., Ishikawa E. (2023). Conservative Treatment of Chronic Subdural Hematoma with Gorei-san. Neurol. Med. Chir..

[11] Chen D., Lin S., Xu W., Huang M., Chu J., Xiao F., Lin J., Peng J. (2015). Qualitative and quantitative analysis of the major constituents in Shexiang Tongxin dropping pill by HPLC-Q-TOF-MS/MS and UPLC-QqQ-MS/MS. Molecules.

[12] Chen J., Yang Y., Shi Y.P. (2011). Simultaneous quantification of twelve active components in Yiqing granule by ultra-performance liquid chromatography: Application to quality control study. Biomed. Chromatogr..

[13] Du K., Yang J., Yang L., Wang Z., Wang R., Shi Y. (2020). Chemical profiling and marker characterization of Huangqin decoction prepared with three types of peony root by liquid chromatography with electrospray ionization mass spectrometry. J. Sep. Sci..

[14] Wang D.D., Liang J., Yang W.Z., Hou J.J., Yang M., Da J., Wang Y., Jiang B.H., Liu X., Wu W.Y. (2014). HPLC/qTOF-MS-oriented characteristic components data set and chemometric analysis for the holistic quality control of complex TCM preparations: Niuhuang Shangqing pill as an example. J. Pharm. Biomed. Anal..

[15] Ministry of Food and Drug Safety of the Republic of Korea (2020). The Korean Pharmacopoeia.

[16] Tian T., Chen H., Zhao Y.Y. (2014). Traditional uses, phytochemistry, pharmacology, toxicology and quality control of *Alisma orientale* (Sam.) Juzep: A review. J. Ethnopharmacol..

[17] Zhao Y.Y. (2013). Traditional uses, phytochemistry, pharmacology, pharmacokinetics and quality control of *Polyporus umbellatus* (Pers.) Fries: A review. J. Ethnopharmacol..

[18] Xu S., Qi X., Liu Y., Liu Y., Lv X., Sun J., Cai Q. (2018). UPLC-MS/MS of Atractylenolide I, Atractylenolide II, Atractylenolide III, and Atractyloside A in Rat Plasma after Oral Administration of Raw and Wheat Bran-Processed Atractylodis Rhizoma. Molecules.

[19] Zou Y.T., Long F., Wu C.Y., Zhou J., Zhang W., Xu J.D., Zhang Y.Q., Li S.L. (2019). A dereplication strategy for identifying triterpene acid analogues in Poria cocos by comparing predicted and acquired UPLC-ESI-QTOF-MS/MS data. Phytochem. Anal..

[20] Liang Y., Li Y., Sun A., Liu X. (2019). Chemical compound identification and antibacterial activity evaluation of cinnamon extracts obtained by subcritical n-butane and ethanol extraction. Food Sci. Nutr..

[21] Seo C.-S., Shin H.-K. (2010). Simultaneous determination of cinnamaldehyde and coumarin in Oryeong-san using HPLC with Photodiode array detector. Herb. Formula Sci..

[22] Xiao S., Hao C., Ai N., Luo K., Wen X., Wang S., Fan X. (2014). Deciphering the differentiations of traditional Chinese medicine analogous formulae by parallel liquid chromatography-mass spectrometry coupled with microplate-based assays. Anal. Methods.

[23] He F., Wang C.J., Xie Y., Cheng C.S., Liu Z.Q., Liu L., Zhou H. (2017). Simultaneous quantification of nine aconitum alkaloids in *Aconiti Lateralis Radix Praeparata* and related products using UHPLC-QQQ-MS/MS. Sci. Rep..

[24] Ji Z., Jiang Y., Lin H., Ren W., Lin L., Guo H., Huang J., Li Y. (2021). Global identification and quantitative analysis of representative components of Xin-Nao-Kang Capsule, a traditional Chinese medicinal formula, by UHPLC-Q-TOF-MS and UHPLC-TQ-MS. J. Pharm. Biomed. Anal..

[25] Tian T., Xu X., Li X., Zhang W., Lu H. (2021). Precision-characterization and quantitative determination of main compounds in Si-Ni-San with UHPLC-MS/MS based targeted-profiling method. J. Pharm. Biomed. Anal..

[26] Jiang Z., Wang X., Wang J., Liu C., Pan J. (2019). Simultaneous determination of eight flavonoids in *Sedum sarmentosum* Bunge from different areas by UHPLC with triple quadrupole MS/MS. Biomed. Chromatogr..

[27] Li X.Y., Xu J.D., Zhou S.S., Kong M., Xu Y.Y., Zou Y.T., Tang Y., Zhou L., Xu M.Z., Xu J. (2018). Time segment scanning-based quasi-multiple reaction monitoring mode by ultra-performance liquid chromatography coupled with quadrupole/time-of-flight mass spectrometry for quantitative determination of herbal medicines: Moutan Cortex, a case study. J. Chromatogr. A.

[28] Kim S.K., Lee S. (2018). Drug-likeness and Oral bioavailability for Chemical compounds of Medicinal Materials Constituting Oryeong-san. Korea J. Herbol..

[29] Yang H., Tuo X., Wang L., Tundis R., Portillo M.P., Simal-Gandara J., Deng J. (2021). Bioactive procyanidins from dietary sources: The relationship between bioactivity and polymerization degree. Trends Food Sci. Technol..

[30] Zhou W., Chen K., Lu Q., Luo Y., Zhang C., Zheng Y., Sha W. (2020). The protective effect of rosavin from *Rhodiola rosea* on radiation-induced intestinal injury. Chem. Biodivers..

[31] Stefanachi A., Leonetti F., Pisani L., Catto M., Carotti A. (2018). Coumarin: A natural, privileged and versatile scaffold for bioactive compounds. Molecules.

[32] Ríos J.L. (2011). Chemical constituents and pharmacological properties of *Poria cocos*. Planta Med..

[33] Wang P., Song T., Shi R., He M., Wang R., Lv J., Jiang M. (2020). Triterpenoids from *Alisma* species: Phytochemistry, structure modification, and bioactivities. Front. Chem..

[34] Chen Q.L., Chen Y.J., Zhou S.S., Yip K.M., Xu J., Chen H.B., Zhao Z.Z. (2018). Laser microdissection hyphenated with high performance gel permeation chromatography-charged aerosol detector and ultra performance liquid chromatography-triple quadrupole mass spectrometry for histochemical analysis of polysaccharides in herbal medicine: Ginseng, a case study. Int. J. Biol. Macromol..

[35] Deshaies S., Sommerer N., Garcia F., Mouls L., Saucier C. (2022). UHPLC-Q-Orbitrap/MS^2^ identification of (+)-Catechin oxidation reaction dimeric products in red wines and grape seed extracts. Food Chem..

[36] Shen Y., Han C., Liu B., Lin Z., Zhou X., Wang C., Zhu Z. (2014). Determination of vanillin, ethyl vanillin, and coumarin in infant formula by liquid chromatography-quadrupole linear ion trap mass spectrometry. J. Dairy Sci..

[37] Yang W., Ye M., Liu M., Kong D., Shi R., Shi X., Zhang K., Wang Q., Lantong Z. (2010). A practical strategy for the characterization of coumarins in Radix Glehniae by liquid chromatography coupled with triple quadrupole-linear ion trap mass spectrometry. J. Chromatogr. A.

[38] Zheng G., Liu M., Chao Y., Yang Y., Zhang D., Tao Y., Zhang J., Zeng C., Wei M. (2020). Identification of lipophilic components in Citri Reticulatae Pericarpium cultivars by supercritical CO_2_ fluid extraction with ultrahigh-performance liquid chromatography-Q Exactive Orbitrap tandem mass spectrometry. J. Sep. Sci..

[39] Tai Y., Zou F., Zhang Q., Wang J., Rao R., Xie R., Wu S., Chu K., Xu W., Li X. (2019). Quantitative analysis of eight triterpenoids and two sesquiterpenoids in Rhizoma alismatis by using UPLC-ESI/APCI-MS/MS and its application to optimisation of best harvest time and crude processing temperature. J. Anal. Methods Chem..

[40] Zhao W., Huang X., Li X., Zhang F., Chen S., Ye M., Huang M., Xu W., Wu S. (2015). Qualitative and quantitative analysis of major triterpenoids in *Alismatis rhizoma* by high performance liquid chromatography/diode-array detector/quadrupole-time-of-flight mass spectrometry and ultra-performance liquid chromatography/triple quadrupole mass spectrometry. Molecules.

[41] Chen L., Qi J., Chang Y.X., Zhu D., Yu B. (2009). Identification and determination of the major constituents in Traditional Chinese Medicinal formula Danggui-Shaoyao-San by HPLC-DAD-ESI-MS/MS. J. Pharm. Biomed. Anal..

[42] Shi Y.Y., Guan S.H., Tang R.N., Tao S.J., Guo D.A. (2012). Simultaneous determination of atractylenolide II and atractylenolide III by liquid chromatography-tandem mass spectrometry in rat plasma and its application in a pharmacokinetic study after oral administration of *Atractylodes macrocephala* rhizoma extract. Biomed. Chromatogr..

[43] Wu L.F., Wang K.F., Mao X., Liang W.Y., Chen W.J., Li S., Qi Q., Cui Y.P., Zhang L.Z. (2016). Screening and analysis of the potential bioactive components of *Poria cocos* (Schw.) wolf by HPLC and HPLC-MS(n) with the aid of chemometrics. Molecules.

[44] Jiang H., Liu J., Wang Y., Chen L., Liu H., Wang Z., Wang B. (2021). Screening the Q-markers of TCMs from RA rat plasma using UHPLC-QTOF/MS technique for the comprehensive evaluation of Wu-Wei-Wen-Tong Capsule. J. Mass Spectrom..

[45] Shu Z., Pu J., Chen L., Zhang Y., Rahman K., Qin L., Zheng C. (2016). *Alisma orientale*: Ethnopharmacology, Phytochemistry and Pharmacology of an Important Traditional Chinese Medicine. Am. J. Chin. Med..

[46] Jang S., Lee A., Hwang Y.H. (2022). Qualitative profiling and quantitative analysis of major constituents in Jinmu-tang by UHPLC-Q-Orbitrap-MS and UPLC-TQ-MS/MS. Molecules.

[47] Center for Biologics Evaluation and Research (CBER) (1996). Guidance for Industry.

